# Unraveling the mechanism of phenol removal from olive mill wastewater using activated olive pomace in semi-industrial fixed-bed system: a DFT and experimental study

**DOI:** 10.1039/d5ra09184e

**Published:** 2026-03-02

**Authors:** Imane Haydari, Achraf Berradi, Elbachir Abddaim, Naaila Ouazzani, Ahmad Hosseini-Bandegharaei, Faissal Aziz

**Affiliations:** a Laboratory of Water Sciences, Microbial Biotechnologies and Natural Resources Sustainability, Faculty of Sciences Semlalia, Cadi Ayyad University BP 2390 40000 Marrakech Morocco i.haydari.ced@uca.ac.ma; b National Center for Research and Studies on Water and Energy (CNEREE), Cadi Ayyad University B. 511 40000 Marrakech Morocco; c Materials, Processes, Environment and Quality Laboratory, Cadi Ayyad University BP 63 46000 Safi Morocco; d Faculty of Chemistry, Semnan University Semnan Iran; e Scientific Research Center, Al-Ayen Iraqi University (AUIQ) Nasiriyah Thi-Qar 64001 Iraq; f Department of Sustainable Engineering, Saveetha School of Engineering, SIMATS Chennai Tamil Nadu 602105 India

## Abstract

This study investigates phenol adsorption from olive mill wastewater (OMW) by a semi-industrial fixed-bed system filled with activated olive pomace (AOP). The adsorption process was modeled with the Homogeneous Surface Diffusion Model (HSDM), accounting for both adsorption equilibrium and mass transfer kinetics. Experiments were conducted at a flow rate of 2 mL min^−1^, a bed height of 47 cm, and an initial phenol concentration of 5693 mg L^−1^. COMSOL Multiphysics 6.0 was used to solve the advection–dispersion equation with linear and Langmuir models, showing good agreement with breakthrough curves. The residual mean square error was less than 0.3, indicating reliable model validation. Key mass transfer coefficients were *k*_f_ = 7.21 × 10^−6^ m s^−1^, *D*_s_ = 2.41 × 10^−6^ m^2^ s^−1^, and *D*_L_ = 3.53 × 10^−1^ m^2^ s^−1^. The adsorption capacity reached 1471 mg g^−1^, demonstrating the effectiveness of olive pomace as a cost-efficient adsorbent for industrial effluents. Recycling and elution tests confirmed the viability of this adsorption technology for large-scale use. Density Functional Theory (DFT) calculations at the B3LYP/6-311G(d,p) level were performed to provide molecular-level insight into the potential active sites and adsorption interactions of the AOP. A techno-economic study was conducted to evaluate the feasibility and economic viability of the project, confirming the validity of the proposed approach for semi-industrial-scale application.

## Abstract 2

This study investigates phenol adsorption from olive mill wastewater (OMW) by a semi-industrial fixed-bed system filled with activated olive pomace (AOP).

## Graphical abstract

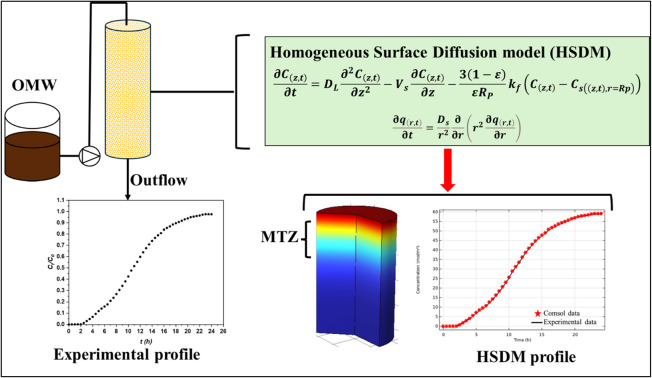

## 1. Introduction

Olive oil production is the mainstay of the agricultural economy in several Mediterranean countries, including Turkey, Morocco, Tunisia, Greece, Spain, and Italy.^1^ Industrial olive oil production has risen in recent years, creating significant amounts of by-products such as liquid waste (olive mill wastewater OMW) and solid waste (olive pomace OP), which represents around 350 kg of OP for every ton of newly picked olives.^2^

The OMW contains high levels of phenolic compounds, suspended organic acids, polysaccharides, lignin, and pectin.^3^ Polyphenols are present in OMW, with concentrations ranging from 600 to 25 000 mg L^−1^.^4^ Polyphenols are toxic pollutants that can cause significant harm to ecosystems and public health if not properly treated. These substances have reached dangerous concentrations due to inappropriate use and release into the environment, generating toxic wastewater that poses considerable health risks.^5^ These hazardous wastes are associated with conditions including liver failure, nausea, protein degradation, cancer, skin lesions, and kidney dysfunction.^6,7^

Numerous studies have investigated the removal and recovery of polyphenols from OMW to assess their economic value and potential negative effects on treatment processes.^8^ Numerous processes have been used to recover phenol, including electrochemical oxidation,^9^ membrane processes,^10^ biological processes,^11^ ion exchange,^12^ distillation,^13^ heterogeneous photocatalysis,^14^ and adsorption.^15^ Despite the advantages of these methods, they also have some disadvantages that limit their large-scale application, such as: high operating cost, production of toxic by-products, high consumption of chemicals, complex operating process, and low efficiency.^6^ On the other hand, many studies indicate that the adsorption process holds great potential for the removal of phenol.^16^ This approach is regarded as beneficial because of its simplicity, effectiveness, moderate energy usage, and low operational expenses.^17^

Unlike many studies that focus on laboratory-scale setups, this work moves towards practical applications by employing a semi-industrial fixed-bed system through mathematical modeling, making the findings more relevant for scaling up in real-world industrial wastewater treatment. The main advantage of mathematical modeling lies in its ability to establish coupling relationships between various physical domains and to efficiently solve them simultaneously.^18^

Chemical engineering processes can be simulated using either commercial software or manually created code. Commercial software such as COMSOL Multiphysics (COMSOL 6.0) has been popular in the chemical engineering sector for decades because of its efficient and user-friendly interface.^19^

Previous studies on OP-based adsorbents for phenolic compound removal have primarily focused on batch adsorption systems and equilibrium analysis,^4^ with limited attention to fixed-bed system performance under semi-industrial conditions. Moreover, most investigations have relied on simplified kinetic models without detailed analysis of intraparticle mass transfer mechanisms.^20^ To date, no study has integrated homogeneous surface diffusion modeling (HSDM) with numerical simulation tools to predict breakthrough behavior and mass transfer zone dynamics for OP-based adsorbents in real OMW treatment systems. Furthermore, although previous studies have employed DFT calculations to investigate adsorption mechanisms at the molecular level, such analyses have rarely been qualitatively correlated with experimental fixed-bed performance.

In this context, the present study proposes a semi-industrial fixed-bed system evaluation of a newly developed AOP adsorbent, coupled with HSDM modeling implemented in COMSOL Multiphysics and complemented by DFT calculations (B3LYP/6-311G(d,p)) to provide qualitative mechanistic insight. This integrated framework enables identification of the dominant mass transport mechanisms, improved predictive capacity for scale-up, and molecular-level interpretation of adsorption behavior. Such a combined experimental-numerical-theoretical approach has not previously been reported for OP-based adsorbents in OMW treatment.

## 2. Materials and methods

### 2.1 AOP

The adsorbent used in this paper was an AOP prepared by.^17^ After washing with distilled water, the pomace was dried in an oven at 105 °C (SF7/S Stuart, UK) for 24 hours. Hydrogen peroxide (H_2_O_2_) was used as a chemical agent to produce an AOP. Dried OP was mixed without H_2_O_2_ 10% solution. The hydrogen peroxide was selected as the activation agent for OP due to its mild and environmentally friendly oxidative properties, which are particularly suitable for lignocellulosic biomass. H_2_O_2_ promotes the partial oxidation of lignin and hemicellulose components, leading to the formation of oxygen-containing functional groups that enhance surface polarity and adsorption affinity toward phenolic compounds.^21^ Unlike conventional chemical activation agents such as KOH, H_3_PO_4_, or ZnCl_2_, hydrogen peroxide does not introduce toxic or corrosive residues and minimizes secondary pollution, which is consistent with the sustainable and green chemistry approach of this work.^22^ The resulting AOP was washed with distilled water and dried in an oven at 60 °C for 24 hours. Techniques and methods for characterizing this adsorbent have been reported previously.^17^

#### 2.1.1 OMW

OMW was collected from a three-phase continuous extraction unit located in Marrakech-Safi region Morocco. It was stored in a plastic drum at room temperature (20–30 °C). The samples collected were subjected to various physic–chemical analyses according to the analytical methods shown in Table 1. Phenolic compounds were quantified using the Folin–Ciocalteu colorimetric method, with caffeic acid as the standard. Absorbance was determined at 760 nm.

**Table 1:** Physicochemical parameters determination

Physicochemical parameters	Method
pH, TDS (total dissolved solids), salinity, EC (electrical conductivity), temperature	Measured by a probe Hanna HI 9829 (Kallang Road, Singapore)
Ammonium	In an alkaline solution, ammonium ions interacted with phenol and hypochlorite to generate indophenol blue, which was spectrophotometrically measured using AFNOR T90-015
Chemical oxygen demand (COD)	A dichromate open reflux technique, as described in AFNOR T90-101, determined the oxygen demand necessary for oxidizing organic matter in a sample *via* a redox reaction
Total phosphorus and *ortho*-phosphates	Spectrometric techniques were employed to assess phosphorus in water, measuring different forms with values larger than 0.01 mg L^−1^, as per AFNOR T90-023
Nitrite	Colorimetric measurement of nitrite ions using AFNOR T90-023 AFNOR T90-013
Phenolic compounds	The Folin–Ciocalteu colorimetric technique^20^ employed caffeic acid as a standard

HPLC-MS/MS analysis was used to determine phenolic substances before and after adsorption was performed. The experiment was undertaken in an Aquity (Waters, CA, USA) with a column oven, an autosampler, and a quaternary pump (HPG). The suggested method used a C18 reversed-phase column (250 × 4.6 mm, 2.6 µm particles) supplied by Thermo Fisher Scientific (CA, USA). Solvent A (0.1%) formic acid aqueous solution and solvent B (methanol) were used to establish a gradient separation. The gradient was created as follows: 0–6 min, linear gradient from 15 to 25% B; 6–12 min, at 25% B; 12–15 min, from 25% to 37% B; 15–20 min, from 37% to 95% B; 20–25 min, returning to initial conditions at 15% B. The flow rate in the mobile phase was 0.5 mL min^−1^. Waters Xevo (US) manufactured the TQD triple quadrupole mass spectrometer outfitted with a heated electrostatic spray (H-ESI) ionization source operating in negative mode. The temperature of the ion transfer tube and the H-ESI vaporizer was set to 250 °C. 3500 V was the electrospray voltage that was set. Complete scan MS capture mode in Q1 (*m*/*z* 50–1000) with fragmentation energy of 20 V.

### 2.2 Methods

#### 2.2.1 Continuous adsorption assays

**Fig. 1:**
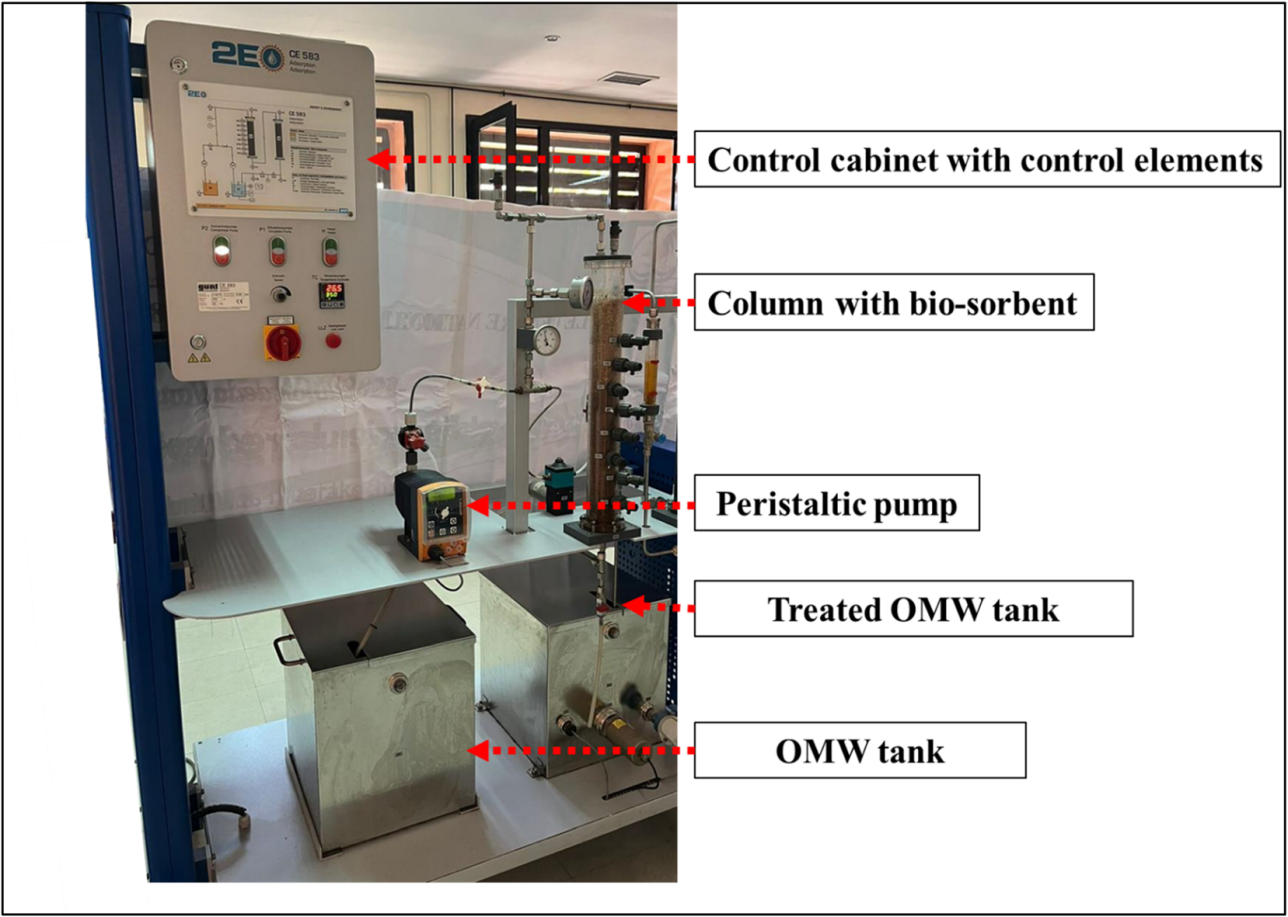
Semi-industrial fixed-bed system.

A semi-industrial fixed-bed system plant with a cubic OMW storage vessel, a peristaltic pump, a 15 cm diameter, and a 47 cm high acrylic column was used (Fig. 1). The AOP (1.40 kg) was introduced into the column. Fixed-bed column tests were carried out for initial total phenol concentrations of 5693 mg L^−1^ (Table 2). The test was carried out at a volumetric flow rate of 2 mL min^−1^. Continuous adsorption experiments were performed at ambient temperature.

**Table 2:** The key operational parameters

Parameters	Value
Flow rate (mL min^−1^)	2
Bed height (cm)	47
Particle size (mm)	3
Temperature (°C)	25
pH	4.98
Initial phenol concentration	5693

Determination of the stoichiometric adsorption capacity (*q*_e_ mg g^−1^), length of the mass transfer zone (MTZ), phenol removal percentage (*R*%), the equilibrium concentration (*C*_e_ mg L^−1^), and treated effluent volume (*V*_eff_ L) was carried out according to eqn (1)–(6).

1 *V*_eff_ = *Qt*_total_

2
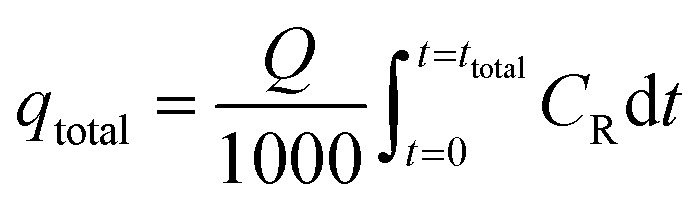
3
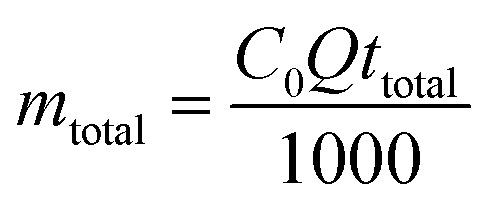
4
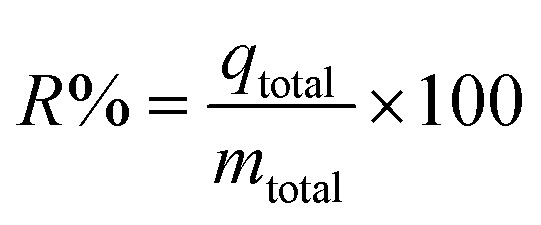
5
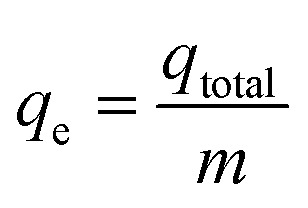
6
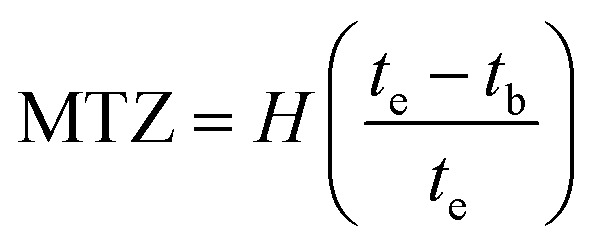
where: *q*_total_ (mg) and *m* total (g) are the quantity of total phenol adsorbed in the column, and that passed through the column, respectively, while *Q* is the volumetric flow rate (mL min^−1^), *C*_0_ (mg L^−1^) is the initial concentration, *H* is the height of the bed-fix (cm), and *t* total time (min). Other experimental parameters are breakthrough time (*t*_b_ min), the mean residence time (*t*_s_ min), and the exhaustion time (*t*_e_ min), defined here as the time required to reach *C*_*t*_/*C*_0_ of 0.1, 0.5, and 0.95, respectively.

### 2.3 Mathematics models

#### 2.3.1 Model conceptualization and formulation

To describe a pollutant in the liquid phase close to the adsorbent grains making up the fixed bed, it is essential to take into account four kinetic resistances to mass transfer:

(i) External transport,

(ii) External diffusion,

(iii) Adsorption to the adsorbent surface,

(iv) Internal surface diffusion.

To better understand this process, models are needed. The film model, introduced by Whitman,^23^ is the most commonly used for external transport. For diffusion inside adsorbent grains, three types of models have been proposed:^24^

- The Homogeneous Surface Diffusion Model (HSDM)^25^ suggests that the sorbate is initially adsorbed onto the outer surface of the grain and subsequently diffuses into the grain, with the porous solid regarded as pseudo-homogeneous.

- The pore diffusion model (PDM)^26^ indicates that the matrix diffuses into the particle pores after passing through the boundary layer, and adsorption occurs only on the inner surface of the particles.

- The pore surface diffusion model (PSDM)^27^ considers that both phenomena, diffusion and adsorption, occur simultaneously.

The HSDM model is commonly quoted in the literature.^28^ It has been used to simulate the adsorption of many organic compounds or ionic species onto adsorbents.^29^

In this work, the HDSM model is based on the following assumptions:

(1) The flow moves in a straight line in one direction only.

(2) Particles act as a uniform mixture where the pollutant propagates.

(3) External mass transfer limitations are considered.

(4) Adsorption equilibrium is mainly observed at the external surface where fluids meet solids.

**Fig. 2:**
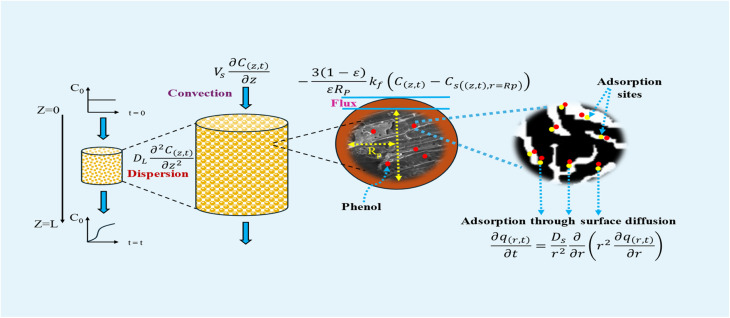
Conceptual framework of the constructed model.

Fig. 2 provides a graphical representation of our model and the adsorption process. To mathematically describe mass transport dynamics within the fixed-bed column, we primarily utilized the convection–dispersion equation, accounting for both diffusion and adsorption within the particles. This model incorporates key operational parameters, such as solution flow velocity, bed height, feed concentration, and adsorbent porosity.

This assumed that surface diffusion is the dominant intraparticle mass transfer mechanism, due to the low void fraction in the adsorbent particles. This assumption is supported by the microporous nature of the particles, which capture phenol *via* the numerous functional groups on the AOP surface. Pore diffusion, which involves phenol molecules moving through the particle's complex pore network, can be neglected in this context. The essential experimental parameters are used to assess the AOP's efficiency in phenol adsorption. However, beyond mere measurement, it is crucial to predict the effluent's behavior as it moves through the system.

Mathematical models are essential for understanding the kinetics of fixed-bed columns and analyzing adsorption breakthrough curves. They provide insight into the mass transfer mechanisms involved, enabling accurate predictions of the process's overall efficiency. This understanding is critical for scaling the process from semi-pilot laboratory experiments to industrial pilot operations and, ultimately, field applications.

This work developed a mathematical model to describe the transport and adsorption of phenol in a fixed-bed column, based on the following assumptions:

• The system operates under constant pressure and temperature conditions.

• Axial dispersion along the column axis, and the flow is uniform over its cross-section.

• The spherical adsorbent particles are uniform in size and density. Moreover, the vacuum proportion in the column remains constant during adsorption.

• Assumed that diffusion at the particle surface is the main mechanism for intraparticle mass transfer. The Langmuir adsorption isotherm describes the equilibrium between solid and liquid.

• The physical properties of the adsorbents and the liquid phase remain stable.

• No chemical reactions take place in the system.

Using the principle of conservation of mass in the liquid phase, it is assumed that:

Accumulation = output − [input + generation due to mass transfer], the one-dimensional convection–dispersion equation can be expressed as follows eqn (7):7
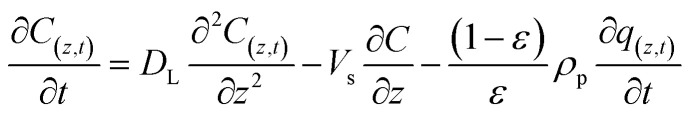

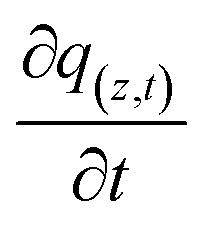
 represents the generation term, being the solid mass transfer rate, and 
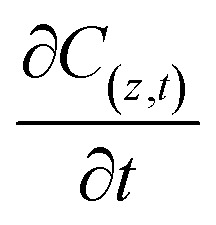
 is the accumulation term in the column bed, *D*_L_ (m^2^ s^−1^) is the axial dispersion coefficient, *z* (m) is the length of the longitudinal axis of the bed, *V*_s_ (m s^−1^) is the interstitial velocity, *t* (h) is the column operating time, and *ε* is the adsorbent porosity.

In addition, the last term in eqn (7) is linked to the mass transfer of phenol molecules driven by the concentration gradient across the stagnant boundary layer of the adsorbent particle eqn (8):8
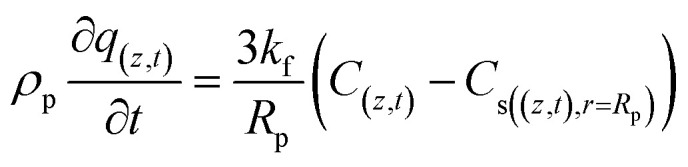
where: *k*_f_ (m s^−1^) is the mass transfer coefficient of the film, *R*_p_ (m) is the particle radius, and *C*_s_ (mg L^−1^) is the concentration of phenol at the surface of the adsorbent particle. Eqn (8) can be substituted in eqn (7) to obtain eqn (9):9
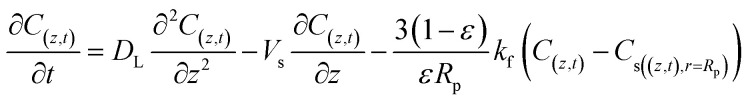


The initial and boundary conditions were applied at both the column inlet and outlet:

10 *C*_(*z*,*t*=0)_ = 0

11
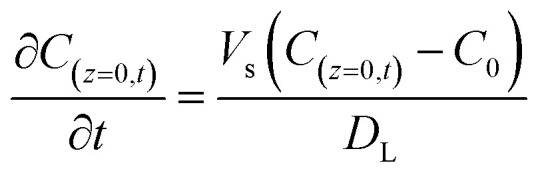
12
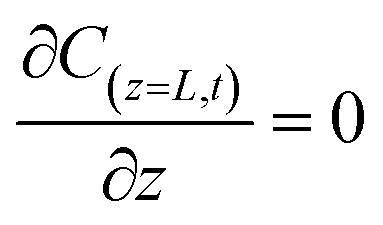


The mass balance equation for the concentration profiles within the adsorbent particle, accounting for surface diffusion in the radial direction, is provided in eqn (13):13
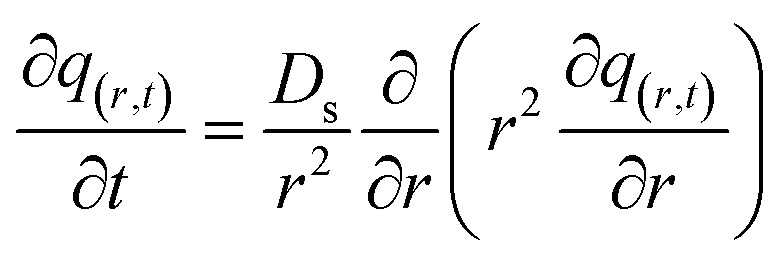
where: *D*_s_ (m^2^ s^−1^) is the surface diffusion coefficient, and *r* (m) is the radial coordinate.

The initial and boundary conditions are provided in eqn (14)–(16):

14 *q*_(*r*,*t*=0)_ = 0

15
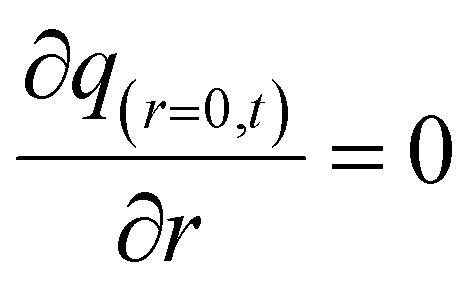

16 *q*_(*r* > 0,*t*)_ = *q*_e_

The equilibrium between the fluid and solid phases at the surface of the adsorbent particle was described using the Langmuir isotherm, as expressed in eqn (17) and calculated in our previous study.^30^17
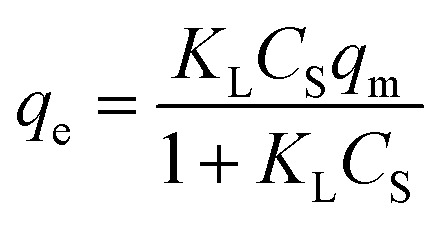
where: *K*_L_ (L mg^−1^) and *q*_max_ (mg g^−1^) are the equilibrium constant and maximum adsorption capacity, respectively.

#### 2.3.2 Determination of the mass-transfer parameters

The model equations that account for axial dispersion, film diffusion, and surface diffusion are commonly referred to as the Homogeneous Surface Diffusion Model (HSDM). For the mass transfer process, empirical correlations utilize dimensionless numbers, such as the intraparticle diffusion coefficient (*D*_s_) – determined from the molecular diffusivity (*D*_*m*_), the external mass transfer coefficient (*k*_f_), the Schmidt number (Sc), the Sherwood number (Sh), the Reynolds number (Re) and the Biot number (Bi) are useful for evaluation eqn (18)–(26):18
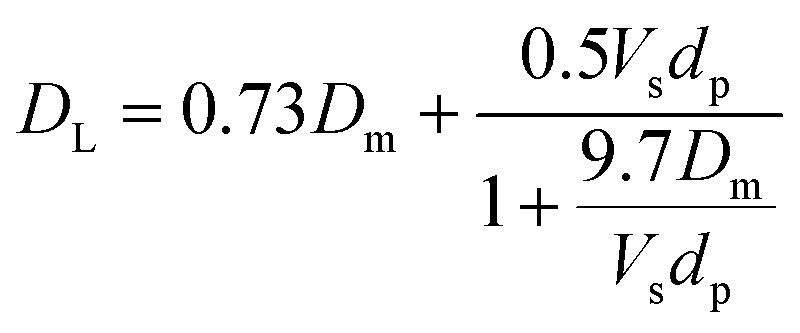
19
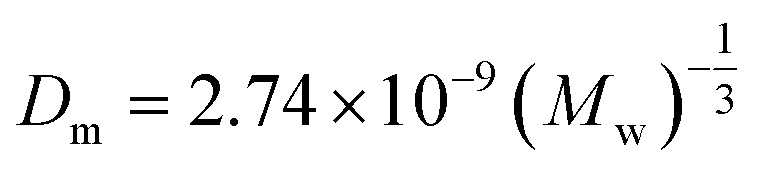
20
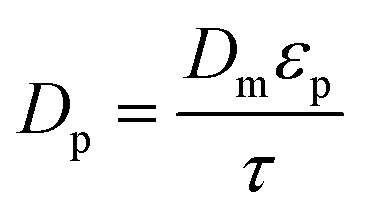
21
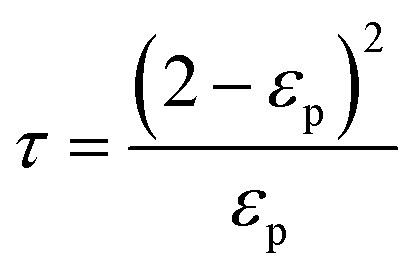
22
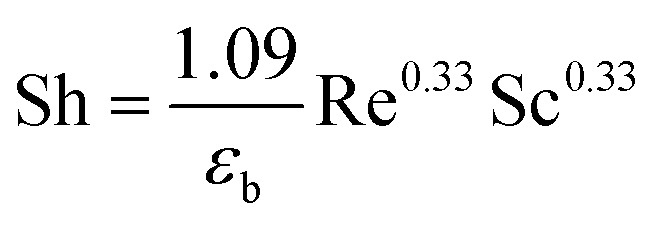
23
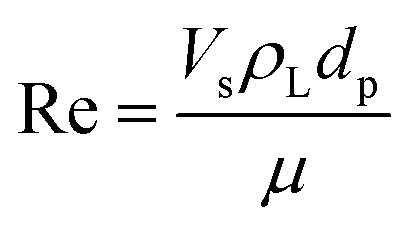
24
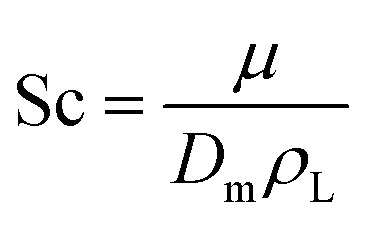
25
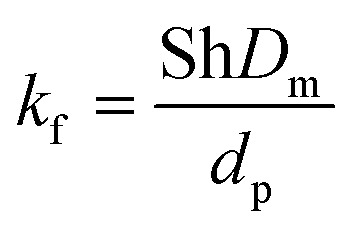
26
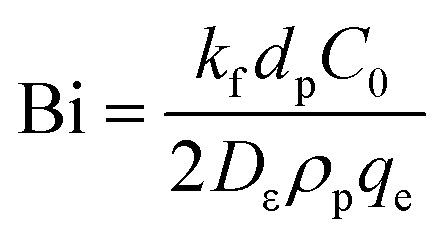
where: *ρ*_L_ (g L^−1^) and *µ* (g m^−1^ s^−1^) denote the density and viscosity of the liquid phase, respectively, *d*_p_ (m) denotes the particle diameter, and *ρ*_p_ (kg m^−3^) is the particle density.

#### 2.3.3 Numerical analysis of the developed model

Adsorption optimization and modeling are powerful tools for predicting performance under real operating conditions. In this study, we utilized COMSOL Multiphysics® version 6.0 to numerically solve the model equations. This interactive environment is designed to model and address a variety of scientific and technical challenges.^30^ Specifically, we employed the Chemical Species Transport module, focusing on the sub-module for mass transport of dilute species through porous media to integrate the convection and diffusion equations within the column domain. The modeling process in COMSOL involves several steps: selecting the appropriate physical interface, creating the geometry using the integrated software or importing it from external sources, and defining the physics, which includes specifying the properties of the adsorbent material and the boundary conditions in the final mesh.

#### 2.3.4 Desorption–regeneration tests

To test the reusability of the AOP, this work carried out four consecutive adsorption and desorption cycles. First, the AOP grains were immersed in a solution of 0.1 M HCl and 10% (v/v) ethanol/water at room temperature. They were then rinsed several times with distilled water. Finally, they were dried at 100 °C before being used for subsequent cycles.

## 3. Results and discussion

### 3.1 OMW characterization

OMW showed that their pH value was acidic, at 4.98 (Table 3), due to the presence of organic acids, including phenolic acids. The values determined in our study are cited in the literature.^20^ The mineral composition of the OMW showed that these wastewaters have a high salt load, especially due to sodium chloride, probably due to the curing process to preserve the olives before crushing, and the natural richness of olives in mineral salts.^31^ This can be explained by the high conductivity values of 12 mS cm^−1^ observed in the OMW. Likewise, the OMW is also heavily polluted by organic matter (expressed as COD). According to Table 3, the values can reach 120 g of O_2_ L^−1^.

**Table 3:** OMW characterization

Parameters	Values
pH	4.98
Orthophosphate (mg L^−1^)	2
Total dissolved solids (TDS) (mg L^−1^)	9514
Salinity (practical salinity unit)	12
Conductivité (mS cm^−1^)	12
Phenolic compounds (mg L^−1^)	5693
Nitrite (mg L^−1^)	0.006
Sulfate (mg L^−1^)	2300
DCO dissout (g of O_2_ per L)	120
Ammonium (mg L^−1^)	50
Phosphore total (mg L^−1^)	4

Another characteristic of OMW is the presence of high concentrations of phenolic compounds. The value measured was 5693 mg L^−1^. Such high concentrations could limit natural bio-degradation and thus cause more or less severe damage to the entire ecosystem.^32^ Nevertheless, the value of this parameter is more or less the same as found in several studies of OMW treatments.^33^

### 3.2 Column adsorption

**Fig. 3:**
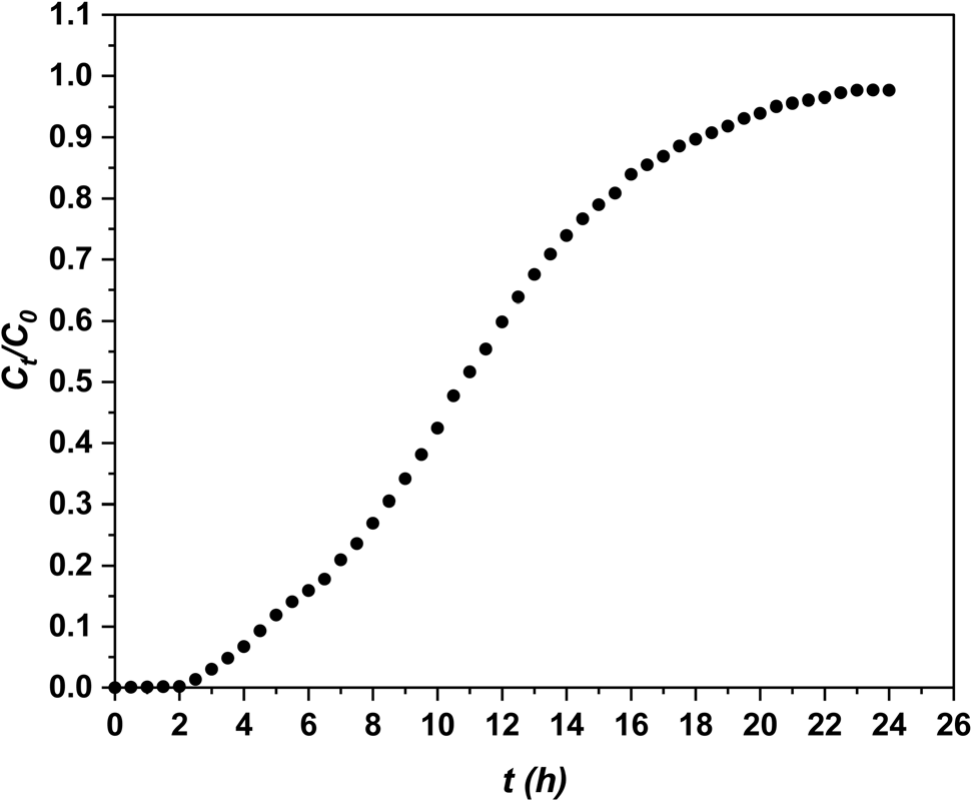
Experimental breakthrough curves for phenol adsorption.

Adsorption tests were conducted for 24 hours at a flow rate of 2 mL min^−1^. *C*_*t*_/*C*_0_*versus* time curves are shown in Fig. 3.

Initially, the upper layers of the fresh adsorbent primarily adsorb phenol, creating the primary adsorption zone. As a result, phenol concentration is low during this stage, leading to very low *C*_*t*_/*C*_0_ values. As the upper layers of the adsorbent become saturated, the primary adsorption zone gradually shifts downward to the cooler granules below. As this zone continues to advance, it eventually allows phenol to break through with the effluent at a specific point, commonly referred to as the breakthrough point. Once the granules are completely exhausted, the *C*_*t*_/*C*_0_ value approaches 1, as illustrated in Fig. 3.

Fig. 3 indicates that the AOP reaches the breakthrough point (*t*_b_) after 3.5 hours. It also highlights the depletion time (*t*_e_) and saturation time (*t*_s_) at 19.5 hours and 12 hours, respectively. The values for the key design parameters of the bed column breakthrough curves for phenol adsorption on the AOP have been calculated and are presented in Table 4. As shown in this table, with an initial concentration (*C*_i_) of 5693 mg^−1^ L and a flow rate (*Q*) of 2 mL^−1^ min, the adsorption capacity (*q*_e_) reaches 1471 mg^−1^ g, while the adsorption efficiency (*R*) is 62% and the mass transfer zone (MTZ) measures 38.56.

**Table 4:** Experimental parameters of the semi-industrial fixed-bed system adsorption

Parameters	Values
*C*_0_ (mg L^−1^)	5693
*Q* (mL min^−1^)	2
*Z* (cm)	47
*V*_eff_	2880
*q*_e_ (mg g^−1^)	1471
*t*_b_ (h)	3.5
*t*_s_ (h)	12
*t*_e_ (h)	19.5
MTZ (cm)	38.56
*R* (%)	62

**Fig. 4:**
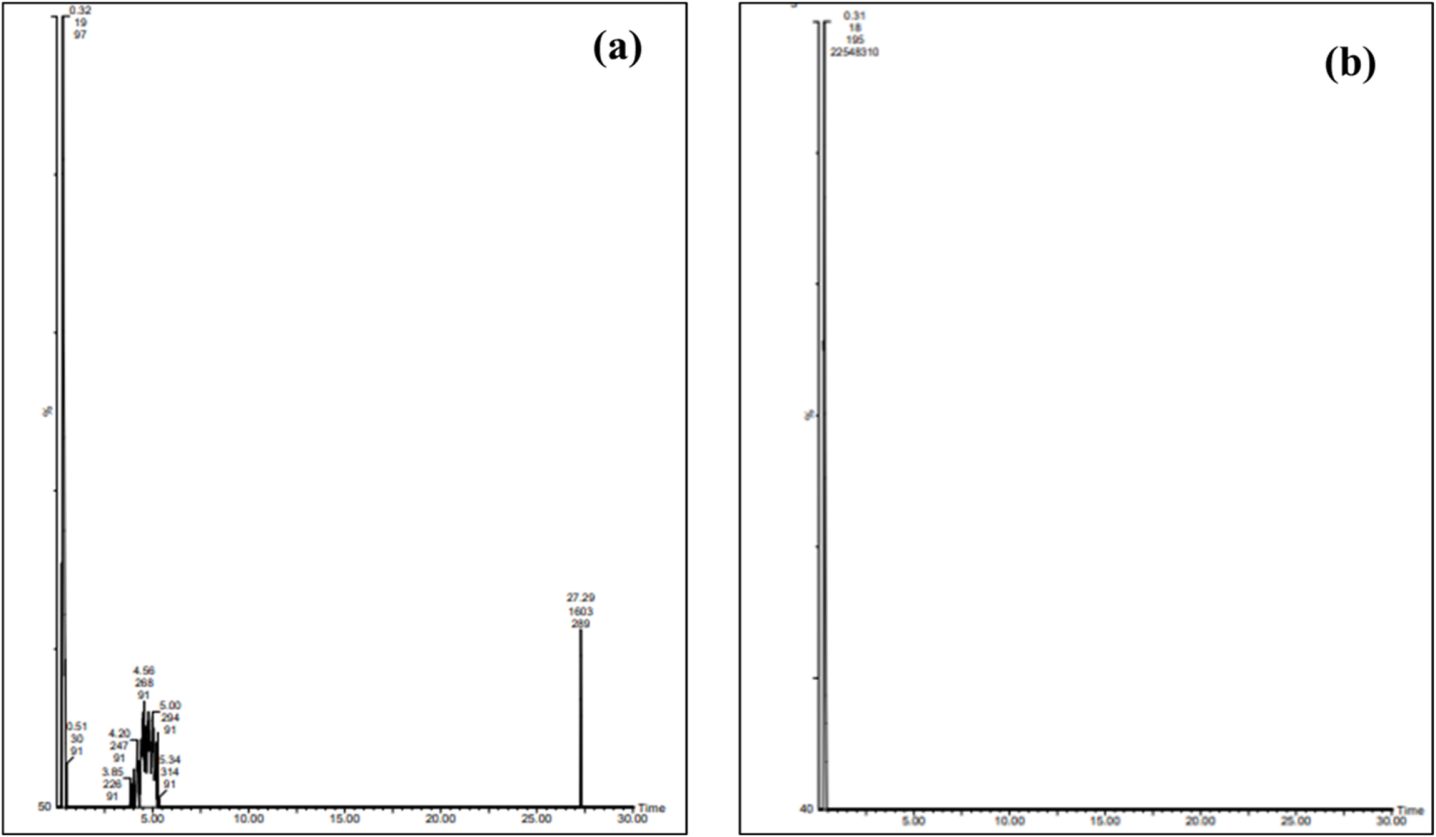
Chromatographic spectrum (LCMSMS): (a) before and (b) after adsorption treatment using a semi-industrial fixed-bed system.

LCMSMS data on the adsorption of phenolic compounds onto AOP grains before and after treatment in a semi-industrial fixed-bed system are shown in Table 5 and Fig. 4(a), (b). A total of nine phenolic compounds were tentatively identified in the OMW, and the analysis of the chromatographic profile revealed the richness of OMW on phenolic compounds (sample 1), like demethyloleuropein as a predominant compound, followed by jaspolyoside. However, the verbascoside, dehydro ligstroside aglycone, oleuropein aglycone derivative, and jaspolyoside derivative presented the lowest quantity in olive mill waste extract. As can be seen in Table 5, we showed the absence of several compounds in sample 2, like pinoresinol, verbascoside, dehydro ligstroside aglycone, oleuropein aglycone derivative, and β-hydroxyacteoside.

**Table 5:** Tentative identification of phenolic compounds in three samples by LCMSMS

No.	RT	*m*/*z* (M − H)^−^	Fragments	Proposed compounds	Molecular formula	1	2
						Area%	
1	0.32	524.98	507.15/377.12	Demethyloleuropein	C_24_H_30_O_13_	55.11	85.12
2	3.85	359.01	307.2	Oleuropein aglycone derivative	C_19_H_20_O_7_	1.3	—
3	4	359.11	297.1	Dehydro ligstroside aglycone	C_19_H_20_O_7_	2.08	—
4	4.2	623.1	606.1	Verbascoside	C_29_H_36_O_15_	3.21	—
5	4.56	639.12	621.1/507.1	β-Hydroxyacteoside	C_29_H_36_O_16_	5.68	—
6	4.8	357.1	—	Pinoresinol	C_20_H_22_O_6_	4.71	—
7	5.03	353.9	—	4-Caffeoylquinic acid	C_16_H_18_O_9_	4.65	—
8	5.34	909.12	507.1	Jaspolyoside derivative	C_42_H_54_O_22_	1.02	—
9	27.29	925.14	507.1	Jaspolyoside	C_42_H_54_O_23_	17.025	tr

### 3.3 Simulation results using COMSOL

COMSOL Multiphysics was utilized to create a numerical model that simulates the experimental data.^34^

HSDM is commonly employed to model breakthrough curves because of its simplicity and accuracy, as emphasized in the study by ref. 30. The coefficients needed for the simulation are presented in Table 6. The values for the parameters *k*_f_, *D*_L_, and *D*_s_ were obtained from the HSDM model applied in the kinetic modeling of adsorption. By incorporating the surface diffusion model, the HSDM effectively characterized the breakthrough curves for phenol adsorption in a downflow fixed-bed system.

**Table 6:** Simulated parameters and mass transfer coefficients of the semi-industrial fixed-bed system adsorption

Parameters	Values
*D*_L_	3.53 × 10^−10^
*D*_s_	2.41 × 10^−6^
*k*_f_	7.21 × 10^−6^
Sc	6.96 × 10^7^
Sh	30.86
Bi	3.32 × 10^−2^

The calculated *D*_L_ value is 3.53 10^−10^ m^2^ s^−1^, as shown in Table 6. Since *D*_L_ is influenced by adsorbent geometry, various theoretical correlations have been utilized to estimate the dispersion coefficient based on the characteristics and mass transport properties of both adsorbents and adsorbates.^30^

The values of *D*_s_ and *K*_f_ were 2.41 × 10^−6^ m^2^ s^−1^ and 7.21 × 10^−6^ cm s^−1^, respectively, calculated from an empirical correlation. In addition, the value of Bi is given. According to Ye *et al.*, 2019,^35^ Bi can be used to distinguish mass transfer regimes: Bi < 0.1 indicates film diffusion control, while Bi > 100 indicates intraparticle diffusion control. In the present system, the results suggest that surface diffusion is likely the predominant mass transfer mechanism under the investigated conditions. This is supported by the nature of the AOP, which has a high external surface area and limited internal porosity, favoring adsorption at the particle surface.^36^ Furthermore, the rapid initial adsorption observed experimentally confirms that surface diffusion dominates. Similar behavior has been reported in the literature for comparable adsorbents.^37^ Therefore, the HSDM, which considers surface diffusion as the dominant transport mechanism, is appropriate for describing the adsorption kinetics in this system.^38^

### 3.4 Model validation

**Fig. 5:**
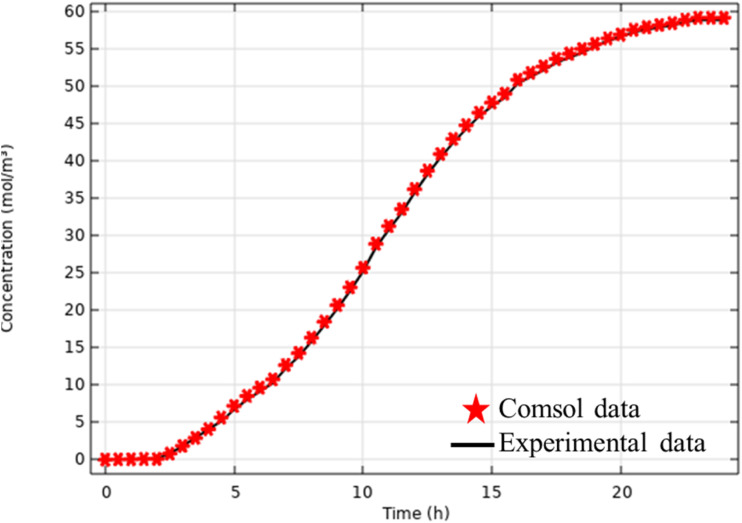
Experimental and simulated breakthrough curves obtained by the Langmuir model using COMSOL (*C*_0_ = 5693 mg L^−1^, *Q* = 2 mL min^−1^).

Fig. 5 shows the breakthrough curves from the simulation and experiment. The simulation curves follow the same trends as the experimental curves. In addition to the visual comparison, an evaluation based on the percentage error (eqn (27)) between simulation and experiment was carried out for two important parameters: adsorption capacity and percentage removal.27
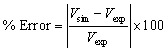
where: *V*_exp_ and *V*_sim_ are the experimental and simulated values.

The experimental total adsorption capacity is 1471 mg g^−1^, while that predicted by the simulation is 1504 mg g^−1^, corresponding to a percentage error of 2.24%, calculated according to eqn (27). Concerning column performance, the simulation predicted a percentage removal of 62% *versus* 63% observed experimentally, corresponding to a percentage error of 1.6%. These results demonstrate the model's accuracy, even under conditions different from those previously studied. The model could also be used for preliminary techno-economic studies without the need for experimentation.

**Fig. 6:**
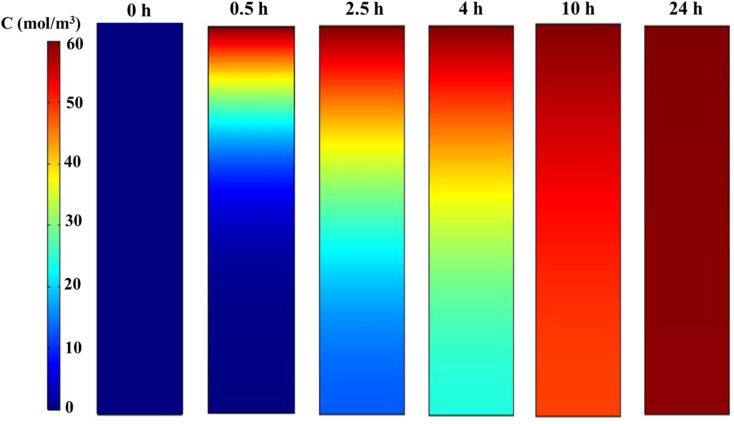
Evolution of phenol concentration profiles using the Langmuir model in COMSOL.

**Fig. 7:**
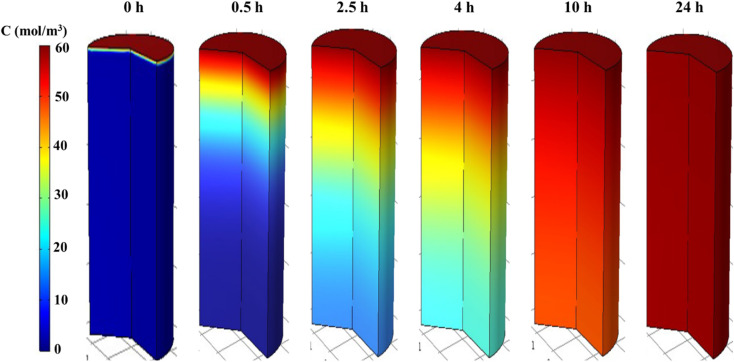
Gradual modification of 3D profiles of axial concentrations at different times, including the initial MTZ formation period from 0 to 0.5 h and the MTZ transfer period from 0.5 to 24 h.

Fig. 6 displays the concentration profiles obtained from the simulation, showing the variation in phenol concentration along the column at a flow rate (*Q*) of 2 mL min^−1^. The predicted concentrations indicate that the AOP in the semi-industrial fixed-bed system continues to absorb phenol molecules until saturation. Fig. 7 provides a direct 3D visualization of the digital data, illustrating the concentration at times of 0, 0.5, 2.5, 4, 10, and 24 hours. During the mass transfer zone (MTZ) formation period from 0 to 0.5 hours, the color of the top layer of the column transitions from dark blue to dark red, indicating complete consumption within approximately 0.5 hours. The MTZ then progresses down the adsorption column, reaching the outlet after 24 hours.

### 3.5 Reusability study

**Fig. 8:**
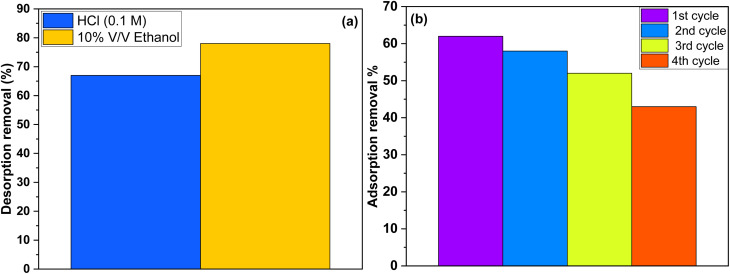
(a) The effect of different regenerating agents on phenol desorption. (b) Four cycles of phenol adsorption on AOP with 10% v/v ethanol as a regenerating agent.

Fig. 8(a) illustrates the impact of HCl and ethanol solutions on phenol desorption from the AOP. The results show that the highest desorption rate, 78%, was achieved with 10% v/v ethanol, as phenol dissolves effectively in ethanol.^39^ Although the pH of the regeneration solution can significantly influence the desorption process by altering the speciation of the adsorbate and the charge distribution on the sorbent surface, using 0.1 M HCl as an eluent resulted in 67% desorption, which is lower than that obtained with the 10% v/v ethanol solution.

Fig. 8(b) presents the results of four cycles of phenol adsorption and desorption using a 10% (v/v) ethanol solution for regeneration. The phenol removal rate dropped from 62% in the first cycle to 43% by the fourth cycle, indicating that non-desorbed phenol molecules gradually saturated the AOP's adsorption sites. The observed decrease in phenol removal efficiency over successive adsorption–desorption cycles indicates that partial adsorption sites on the surface of the AOP may become blocked or altered during regeneration. To address this limitation, further studies should focus on optimizing different desorption agents and conditions in order to identify the most effective approach for the complete removal of phenolic compounds from the adsorbent surface. Such optimization would enhance the long-term reusability of AOP in continuous treatment systems and ensure sustained high adsorption performance.^20^

### 3.6 Theoretical study and adsorption mechanism

DFT is widely used to analyze the electronic and geometric structures of molecules. The calculation of vibrational frequencies plays an important role in theoretical investigations of organic compounds. In addition, DFT provides an effective framework for exploring the relationship between molecular geometry and electronic properties.^40,41^

**Fig. 9:**
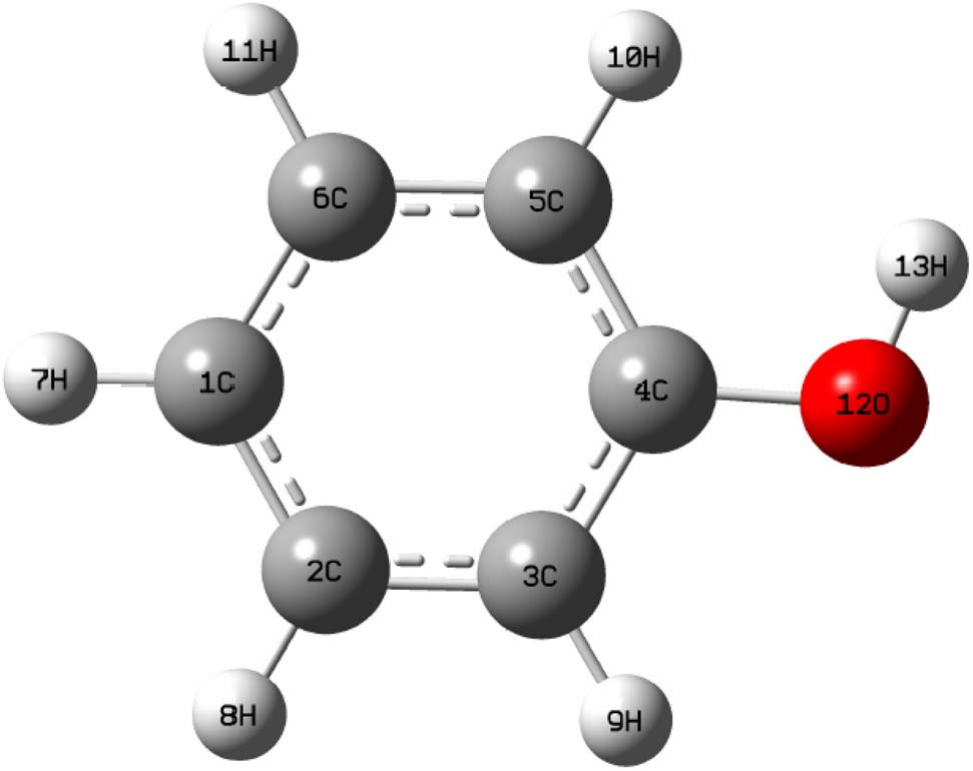
The optimized molecular structure and atom numbering scheme adopted in this study for phenol at the B3LYP/6-311G(d,p).

The nucleophilic Parr function (*P*_K_^−^) and electrophilic Parr function (*P*_K_^+^) were determined through Mulliken analysis of the atomic spin density in nucleophilic and electrophilic molecules. The *P*_K_^−^ and *P*_K_^+^ values for phenol were calculated using the DFT B3LYP/6-311G(d,p) method (Fig. 9).^40^ The electrophilicity and nucleophilicity of phenol are determined by the following eqn (28) and (29):

28 *ω*_K_ = *ω* × *P*_K_^+^

29 *N*_K_ = *N* × *P*_K_^−^

**Fig. 10:**
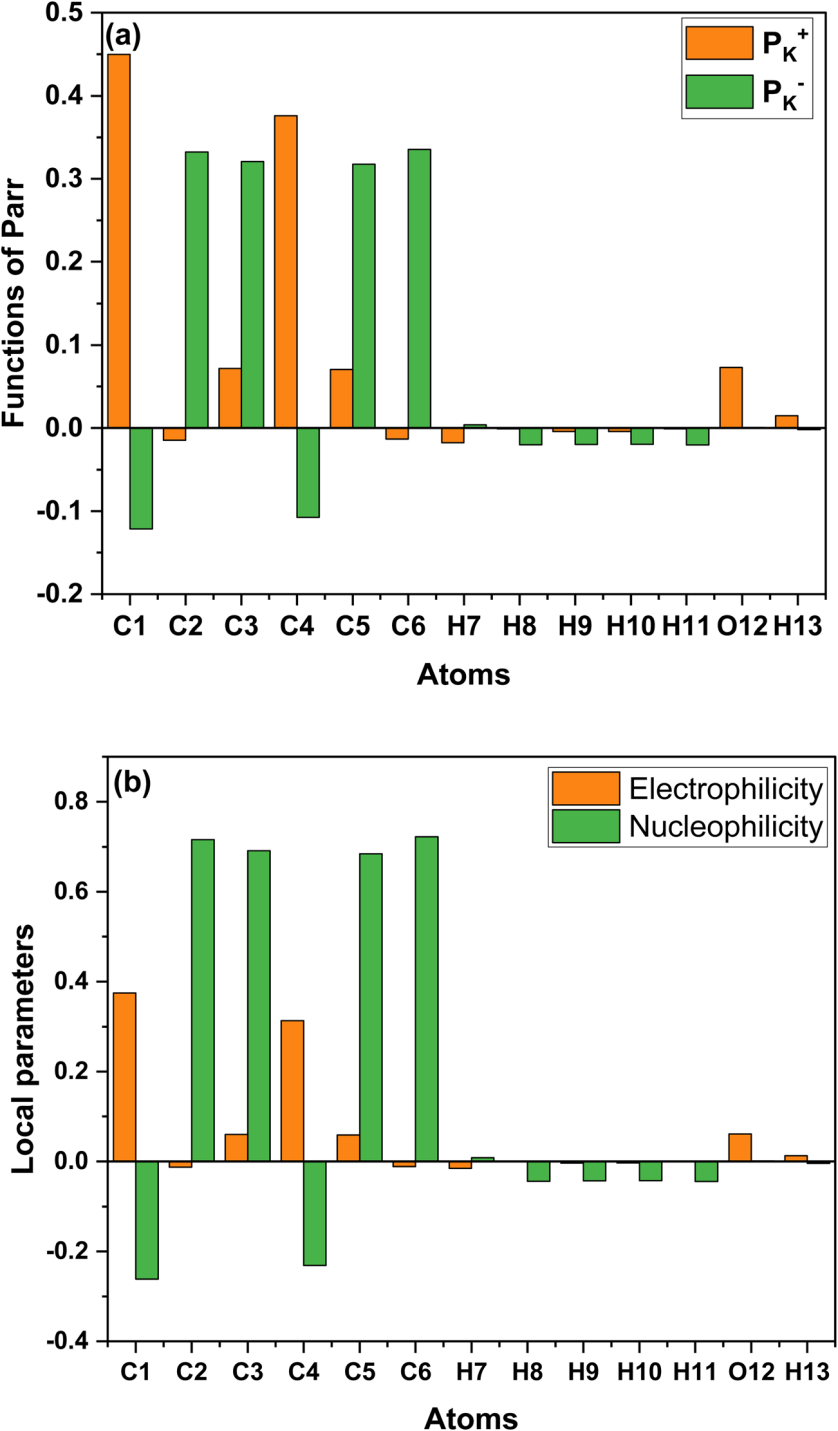
The distribution of the functions of Parr *P*_K_^+^ and *P*_K_^+^ (a) and the electrophilicity and nucleophilicity (b).

The values of *P*_k_^+^, *P*_k_^−^, *ω*_k_, and *N*_k_ calculated using the DFT B3LYP/6-311G(d,p) method are presented in Fig. 10(a) and (b). Due to their polarization and high electronegativity, certain carbon (C) atoms serve as excellent adsorption sites. Atoms with π-bonds demonstrate high reactivity.^40^ However, the Parr parameter plots suggest that carbon atoms can function as both electrophilic and nucleophilic centers when interacting with the adsorbent surface, due to charge dispersion around these atoms. Specifically, atoms C2, C3, C5, and C6 act as nucleophilic centers with high *N*_k_ values of 0.722, while atoms C1 and C4 act as electrophilic centers, with *ω*_k_ values around 0.375.

**Fig. 11:**
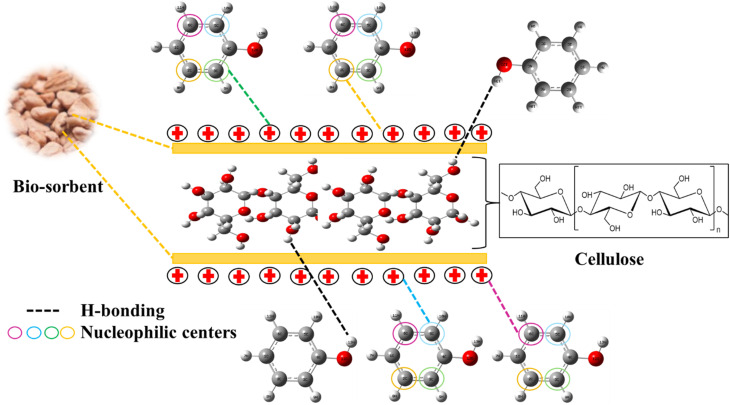
Adsorption mechanism.

To conclude the mechanism of phenol adsorption on the AOP (Fig. 11), it is necessary to collate the results. According to these results, the AOP surface has a high adsorption affinity. XRD spectrum analysis^17^ revealed the presence of the cellulose structure. The presence of –OH hydroxyl groups on the surface of the AOP enables the formation of hydrogen bonds. On the other hand, the theoretical study of phenol in its stable state shows that the molecule possesses nucleophilic centers, represented by carbon atoms C2, C3, C5, and C6, which are oriented towards the positive surface of the AOP (according to pH_zpc_ = 8.64).^17^

### 3.7 Comparative study

The comparative analysis presented in Table 7 clearly demonstrates the superior performance of the AOP developed in this study, which achieved an adsorption capacity of 1471 mg g^−1^ for phenolic compounds. This unusually high value is likely influenced by the very high inlet concentration, the presence of multiple phenolic species, and the fixed-bed integration method. While not necessarily incorrect, it should be interpreted as an apparent system capacity rather than an intrinsic equilibrium adsorption capacity, and its exceptional magnitude requires contextualization relative to the literature. Indeed, this value is substantially higher than those reported for other olive-derived adsorbents, including olive pomace biochar (309.1 mg g^−1^), activated olive pomace reported by Rocha *et al.* (163 mg g^−1^), raw olive pomace (66.67 mg g^−1^), and olive stone (122 mg g^−1^), as well as conventional activated carbon (78 mg g^−1^). The markedly enhanced adsorption capacity obtained in this work can be attributed to the activation strategy and optimized textural and surface properties of the adsorbent, which likely promoted stronger interactions with phenolic compounds, such as π–π interactions, hydrogen bonding, and electrostatic attractions. Compared to raw and biochar-based materials, the AOP used here benefits from a higher density of accessible active sites and improved pore structure, facilitating more efficient mass transfer and adsorption.^8^

**Table 7:** Adsorption capacities of phenolic compounds from OMW onto various adsorbents

Adsorbent	Adsorption capacity (mg g^−1^)	References
Olive pomace biochar	309.1	42
Activated olive pomace	163	8
Olive pomace	66.67	2
Olive stone	122	43
Activated carbon	78	44
AOP	1471	This study

Overall, this comparative study highlights the added value of waste-derived AOP as a highly efficient and sustainable adsorbent for phenolic compound removal, outperforming both unmodified olive residues and commercial activated carbon. These results support its strong potential for practical application in OMW treatment, particularly in high-load systems where elevated adsorption capacity is required.^1^

### 3.8 Techno-economic aspect of this work

An economic analysis using the ROR method was performed to evaluate the effectiveness of AOPs for phenol removal from OMW in a semi-industrial fixed-bed system. Table 8 provides details of the semi-industrial fixed-bed system OMW treatment. The annual costs include installation, maintenance, operation, replacement, and indirect expenses. The ROR of the AOP^45^ is determined when the present value of the investment reaches zero (Table 8). The ROR for each project can be calculated as follows (eqn (30)):30
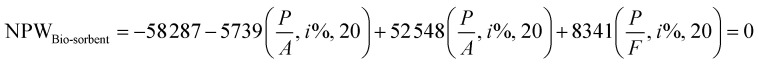


**Table 8:** Economic details of a semi-industrial fixed-bed column using AOP prepared from OP

Costs and incomes	AOP
Initial investment ($)	58 287
Annual cost ($)	5739
Annual income ($)	52 548
Salvage value ($)	8341
Useful life (year)	20
MARR	15

Additionally, projects can be evaluated by comparing the Rate of Return (ROR) with the Minimum Attractive Rate of Return (MARR). A project is economically accepted if the ROR is greater than or equal to the MARR, and it is rejected if the ROR is less than the MARR.^46^ Solving the equation yields an ROR of 80%, indicating that projects using AOPs as adsorbents are economically viable, surpassing the 15% threshold. This economic analysis using the ROR method shows that AOPs offer a cost-effective, versatile, environmentally friendly, and high-capacity solution for phenol removal.^47^

### 3.9 Implications of this work

The study has several notable implications for the body of knowledge, the economy, and society. This work deepens the understanding of how AOPs derived from agricultural by-products, like OP, can be applied to wastewater treatment. By focusing on the mass transfer mechanism in a semi-industrial fixed-bed system, the study contributes to advancing adsorption science, particularly for phenol removal. The use of the Homogeneous Surface Diffusion Model (HSDM) in this work bridges the gap between theoretical adsorption models and real-world applications.^48^

The integration of numerical simulation using COMSOL and validation with experimental breakthrough curves also offers a methodological framework for future work. The study also incorporates DFT calculations to qualitatively examine potential adsorption sites, thereby providing molecular-level insight into the interactions between phenol and AOP. This contributes to the broader field of environmental chemistry and sustainable wastewater treatment technologies.^49^

From an economic perspective, the work highlights the potential for valorizing OMW, specifically OP, as a low-cost AOP. The study offers a cost-effective method for treating industrial wastewater. OP, a by-product of olive oil production, is an abundant and inexpensive material that could be utilized as a low-cost adsorbent. By transforming agricultural waste into a resource for environmental remediation, this approach could reduce the costs associated with wastewater treatment for industries. Additionally, it provides a potential revenue stream for olive oil producers by turning their waste into a valuable product, promoting a circular economy model.^4^

The societal importance of this work also lies in its potential to improve public health and environmental quality. The untreated discharge of phenol-rich OMW poses risks to freshwater systems, agricultural lands, and communities. By offering a viable solution for treating OMW, the study contributes to the protection of natural water resources and the reduction of health hazards. The use of eco-friendly, bio-based adsorbents also aligns with the global push for more sustainable, low-impact technologies that can support societal efforts to achieve environmental sustainability and well-being.^50^

### 3.10 Limitations of this work and mitigation strategies

The study has several limitations, although mitigation strategies can help address them. One of the primary limitations is the scale of the experimental setup. While the fixed-bed column used is classified as semi-industrial, scaling this system up to full industrial levels may present challenges. The adsorption dynamics in a larger-scale operation could be different, and results obtained from the semi-industrial setup may not be directly applicable to industrial plants. A potential mitigation strategy is to perform pilot-scale testing as an intermediate step between laboratory and full-scale application. This would provide better insights into how the process performs at larger scales and identify any potential design adjustments needed.^30^

Another limitation lies in the use of a homogeneous surface diffusion model (HSDM) to describe the mass transfer processes. While the model has proven effective in representing equilibrium and kinetics in smaller systems, it may not capture all the complexities of real-world wastewater with variable compositions. The limitation could be mitigated by complementing the HSDM with other advanced models or integrating real-time data monitoring systems to adjust operational parameters in response to fluctuating wastewater characteristics.^30^

The adsorbent regeneration process is another challenge, as repeated cycles of adsorption and desorption might lead to the degradation of the AOP's structural integrity, reducing its efficiency over time. Although this limitation affects the long-term feasibility of using OP in continuous applications, it can be mitigated by performing regeneration studies with enhanced desorption, such as optimizing pH or temperature, to improve the longevity of the adsorbent.^51^

Lastly, the study focuses on phenol removal, but real OMW contains a mixture of organic compounds, each of which may behave differently during adsorption. This limitation can be addressed by conducting multi-component adsorption studies to better understand how the presence of other substances affects phenol removal. A more holistic approach would be to combine adsorption with other technologies, creating a hybrid system that can deal with a wider range of contaminants more efficiently. By addressing the limitations through further testing, scaling strategies, advanced modeling, and combined treatments, its application can be optimized for large-scale, real-world conditions.^34^

The observed decrease in phenol removal efficiency over successive adsorption–desorption cycles suggests that partial blockage or modification of active sites may occur during regeneration.^20^ However, detailed physicochemical characterization of the AOP after regeneration was not performed in the present study, which limits definitive conclusions regarding structural or surface alterations. Therefore, future investigations should include post-regeneration analyses (*e.g.*, FTIR, SEM) to better elucidate the mechanisms responsible for performance decline and to optimize desorption conditions. Such efforts would contribute to improving the long-term stability and reusability of AOP in continuous treatment systems.

## 4. Conclusion

This study presents innovative simulations conducted with COMSOL software, offering a crucial theoretical foundation for analyzing and optimizing the adsorption process on a semi-industrial scale. The HSDM model has been successfully validated using experimental breakthrough data from AOP for phenol removal from OMW.

The model was also utilized to determine essential mass transfer parameters, including axial dispersion, surface diffusion, and film transfer. The mass transfer coefficients *k*_f_, *D*_s,_ and *D*_L_ obtained are 7.21 × 10^−6^ m s^−1^, 2.41 × 10^−6^ m^2^ s^−1^, and 3.53 × 10^−10^ m^2^ s^−1^, respectively. Intraparticle diffusion dominates the adsorption mechanism. At the same time, the maximum capacity reached 1471 mg g^−1^. In the study of adsorbent repeatability, the phenol removal rate by the AOP dropped from 62% in the first cycle to 43% in the fourth cycle. In addition, semi-industrial pilot-scale trials are required to design a large-scale adsorption system using the AOP.

Modeling results facilitate implementation and enable significant advancements in the removal of phenol from OMW within a short timeframe. Consequently, the time and costs associated with large-scale designs are significantly reduced, as demonstrated by the economic analysis using the ROR method. This underscores the AOP's cost-effectiveness, environmental friendliness, versatility, and high capacity. Additionally, DFT calculations can assist in the qualitative interpretation of experimental observations by identifying potential reactive sites that may contribute to phenol adsorption. Moreover, theoretical approaches provide a useful framework for exploring molecular interaction behavior in aqueous environments.

## Author contributions

Imane Haydari is responsible for writing original draft; writing-review and editing; Achraf Berradi is responsible for conceptualization; Elbachir Abddaim is responsible for methodology; Naaila Ouazzani is responsible for conceptualization; Ahmad Hosseini-Bandegharaei is responsible for writing-review and editing; Faissal Aziz is responsible for supervision.

## Conflicts of interest

The authors declare that they have no known competing financial interests or personal relationships that could have appeared to influence the work reported in this paper.

## Data Availability

Data available on request from the authors.
